# Parameter Selection and Performance Comparison of Particle Swarm Optimization in Sensor Networks Localization

**DOI:** 10.3390/s17030487

**Published:** 2017-03-01

**Authors:** Huanqing Cui, Minglei Shu, Min Song, Yinglong Wang

**Affiliations:** 1 Shandong Province Key Laboratory of Wisdom Mine Information Technology, Shandong University of Science and Technology, Qingdao 266590, China; 2 Shandong Provincial Key Laboratory of Computer Networks, Shandong Computer Science Center (National Supercomputer Center in Jinan), Jinan 250101, China; shuml@sdas.org (M.S.); wangyl@sdas.org (Y.W.); 3 Computer Science Department, Michigan Technological University, Houghton, MI 49931, USA; mins@mtu.edu

**Keywords:** wireless sensor networks, particle swarm optimization, localization, parameter selection, performance comparison

## Abstract

Localization is a key technology in wireless sensor networks. Faced with the challenges of the sensors’ memory, computational constraints, and limited energy, particle swarm optimization has been widely applied in the localization of wireless sensor networks, demonstrating better performance than other optimization methods. In particle swarm optimization-based localization algorithms, the variants and parameters should be chosen elaborately to achieve the best performance. However, there is a lack of guidance on how to choose these variants and parameters. Further, there is no comprehensive performance comparison among particle swarm optimization algorithms. The main contribution of this paper is three-fold. First, it surveys the popular particle swarm optimization variants and particle swarm optimization-based localization algorithms for wireless sensor networks. Secondly, it presents parameter selection of nine particle swarm optimization variants and six types of swarm topologies by extensive simulations. Thirdly, it comprehensively compares the performance of these algorithms. The results show that the particle swarm optimization with constriction coefficient using ring topology outperforms other variants and swarm topologies, and it performs better than the second-order cone programming algorithm.

## 1. Introduction

Wireless sensor networks (WSNs) are an important infrastructure of the Internet of Things used for sensing the surrounding information, whose applications can be classified into monitoring and tracking in the fields of military and public [1]. In these applications, spatial information is one of the most important contexts of the sensed data, and the location information can support the coverage, routing, and many other operations of a WSN. However, the sensor nodes of a WSN are usually deployed in an ad hoc manner without any prior knowledge of their locations, so it is essential to determine the node’s location, which is referred to as *localization*.

A possible solution is to equip each sensor node with a global positioning system (GPS) device, but it is not suitable for large-scale deployment due to the constraints of cost and energy. Hence, only a part of sensor nodes (named *anchors*) are equipped with GPS devices. These anchors serve as references to the other nodes (named *unknown nodes*), which are to be localized. There exist well-organized overviews of sensor localization algorithms [2,3]. Localization consists of ranging and estimation phases. In the ranging phase, the nodes measure their distances from anchors using received signal strength, time of arrival, time difference of arrival, link quality indicator, or angle of arrival. In the estimation phase, the node’s position is estimated based on the ranging information.

A popular estimation approach is to formulate the localization problem as an optimization problem, and then use an optimization algorithm to solve the problem. Traditional optimization algorithms are widely used in localization [4,5,6,7], such as least square, maximum-likelihood, semi-determined programming, and second-order cone programming. Recently, soft computing algorithms have been widely applied to solve this problem [8,9], such as Cuckoo search algorithm [10], artificial neural network [11], bacterial foraging algorithm [12], bat algorithm [13], and biogeography-based optimization [9,14].

Particle swarm optimization (PSO) is also an important soft computing algorithm which models the behavior of a flock of birds. It utilizes a population of *particles* to represent candidate solutions in a search space, and optimizes the problem by iteration to move these particles to the best solutions with regard to a given measure of quality. Compared with the above algorithms, the advantages of particle swarm optimization are the following [15,16,17].

1. Ease of implementation on hardware or software.
2. High-quality solutions because of its ability to escape from local optima.
3. Quick convergence.

Recently, PSO has been used in many issues of WSNs [17,18]. This paper focuses on the PSO-based localization algorithms for static WSNs, where all sensors are static after deployment. Some PSO-based localization algorithms with different population topologies are compared in Cao et al. [19]. However, they do not consider the recently-proposed PSO-based localization algorithms, nor do they give parameter selections. The existing PSO’s parameter selection guidelines [16] are not based on the objective function in localization problem of WSN, so these parameters cannot achieve the optimal localization performance.

The main contributions of this paper are as follows.

1. It surveys the popular particle swarm optimization variants and particle swarm optimization-based localization algorithms for wireless sensor networks.
2. It presents parameter selection of nine particle swarm optimization variants and six types of swarm topologies by extensive simulations.
3. It comprehensively compares the performance of these algorithms.

The rest of the paper is organized as follows. The localization problem and PSO are introduced in Section 2. Section 3 surveys the PSO-based localization algorithms, and Section 4 presents their parameter selections. Section 5 compares the performance of PSO-based localization algorithms. Section 6 concludes the paper and presents the future work.

## 2. Statements of Localization Problem and PSO

### 2.1. Localization Problem for Static WSNs

A WSN is a network of *N* sensor nodes, including $N_{A}$ anchors and $N_{U}$ unknown nodes, where $N_{A}\ll N_{U}$. The WSN is deployed in a two-dimensional region of interest at random. The region is often assumed as a square of side-length *L*. Suppose all the sensor nodes have the same communication range, which is a circle of radius *R*. Two sensor nodes are called *neighbors* if one of them lays in the communication range of the other, and the distance between them can be measured. Obviously, an unknown node can be localized if it has at least three neighboring anchors.

Since $N_{A}\ll N_{U}$, the localization of static WSNs is an iterative procedure. The unknown nodes with at least three neighboring anchors are localized first, and then the other unknown nodes are localized based on the information of neighboring anchors and localized unknown nodes iteratively. Both the anchors and localized unknown nodes are called *reference nodes* in this paper.

During localization, suppose an unknown node $U_{j}$ ($j=1,2,\cdots ,N_{U})$ has *n* neighboring reference nodes. Let ${\hat{d}}_{kj}$ be the measured distance between $U_{j}$ and reference node $B_{k}$ (k=1,2,⋯,n), $\left({\hat{x}}_{u_{j}},{\hat{y}}_{u_{j}}\right)$, and $\left(x_{u_{j}},y_{u_{j}}\right)$ be the estimated and actual positions of $U_{j}$, and $\left(x_{b_{k}},y_{b_{k}}\right)$ be the position of $B_{k}$. Then, the localization result should satisfy

(1) $${\hat{d}}_{kj}=\sqrt{{\left({\hat{x}}_{u_{j}}-x_{b_{k}}\right)}^{2}+{\left({\hat{y}}_{u_{j}}-y_{b_{k}}\right)}^{2}}\;.$$

The distances measured by any ranging method are inaccurate, so it is impossible to find an accurate solution to (1). Let ${\tilde{d}}_{kj}$ be the right part of (1), and be referred to as *estimated distance*. Obviously,

(2) $${\tilde{d}}_{kj}\neq {\hat{d}}_{kj}$$

because of the inaccurate measured distances. Then, the purpose of localization is to minimize the difference between ${\hat{d}}_{kj}$ and ${\tilde{d}}_{kj}$, which is

(3) $$f_{1}=\sum_{k=1}^{n}{\left({\hat{d}}_{kj}-{\tilde{d}}_{kj}\right)}^{2}.$$

For most ranging techniques, the measurement error is related to the distance between the two sensor nodes, and larger distances cause larger error [20]. Hence, weight $w_{k}$ is introduced so that the nearer neighboring reference nodes a play greater role in localization, as shown in (4).

(4) $$f_{2}=\sum_{k=1}^{n}w_{k}{\left({\hat{d}}_{kj}-{\tilde{d}}_{kj}\right)}^{2}\;,$$

where $w_{k}$ is the weight of the neighboring reference node $B_{k}$ of $U_{j}$, defined as

(5) $$w_{k}=\frac{\frac{1}{{\hat{d}}_{kj}}}{\sum_{k=1}^{n}\frac{1}{{\hat{d}}_{kj}}}\;.$$

Besides, the following equations are also discussed in related literature:

(6) $$f_{3}=\frac{1}{n}f_{1}$$

(7) $$f_{4}=\frac{1}{n}f_{2}$$

which are, respectively, the average of $f_{1}$ and $f_{2}$ on number of neighboring reference nodes *n*.

Equations (3), (4), (6) and (7) are called *objective functions*.

### 2.2. Particle Swarm Optimization

The PSO-based localization algorithm uses PSO to solve one of the above objective functions. Because the WSN considered in this paper is in a two-dimensional region, the search space of the PSO is constrained to two-dimensions.

Let *M* be the number of particles of the PSO. Particle *i* occupies three two-dimensional vectors $P_{i}$, $Q_{i}$, and $V_{i}$, representing its current location, previous best position, and current velocity. Besides, Gb denotes the position of the best particle so far. In each iteration, particle *i* updates its position and velocity according to the following equations.

(8) $$\begin{array}{cc} \begin{array}{c} V_{ij}=\omega V_{ij}+c_{1}\varphi_{1j}\left(Q_{ij}-P_{ij}\right)+c_{2}\varphi_{2j}\left(Gb_{j}-P_{ij}\right)\\ P_{ij}=P_{ij}+V_{ij} \end{array} & j=1,2, \end{array}$$

where $c_{1}$ and $c_{2}$ are cognitive and social acceleration coefficients, respectively, $\varphi_{1j}$ and $\varphi_{2j}$ are random numbers uniformly distributed in (0,1), and *ω* is the inertia weight. $c_{1}$ propels the particle towards the position where it had the best fitness, while $c_{2}$ propels the particle towards the current best particle. The stochastic $V_{i}$ may become too high to keep all particles in the search space. Hence, $V_{max}$ is introduced [16] to bound $V_{ij}$ within the range $\left[-V_{max},V_{max}\right]$.

*ω* is an important parameter. Linearly decreasing and simulated annealing types are the best ones of all adjustment methods [21]. Due to the computational and memory constraints of sensor nodes, the linearly decreasing method is adopted in many PSO-based localization algorithms, which is

(9) $$\omega =\omega_{max}-\frac{\omega_{max}-\omega_{min}}{t_{max}}\times t$$

where $t_{max}$ is the maximum number of allowable iterations, $\omega_{max}$ and $\omega_{min}$ are maximum and minimum weights, respectively, and *t* is the current iteration.

As we can see, PSO needs each particle to communicate/connect with the other particles to obtain Gb. The connections among particles are called *topology*. There are two kinds of topologies: *global-best* and *local-best*. The former allows each particle to access the information of all other particles, and the latter only allows each particle to access the information of its neighbors according to different local-best topology [22,23]. Because each particle has a different swarm of neighboring particles in its local-best topology, this topology ensures that the particles have full diversity. Local-best topology uses $Lb_{i}$ instead of Gb to represent the best position of the neighboring particles of particle *i*, and its update function is:

(10) $$\begin{array}{cc} \begin{array}{c} V_{ij}=\omega V_{ij}+c_{1}\varphi_{1j}\left(Q_{ij}\;-\;P_{ij}\right)+c_{2}\varphi_{2j}\left(Lb_{ij}\;-\;P_{ij}\right)\\ P_{ij}=P_{ij}+V_{ij} \end{array} & j=1,2. \end{array}$$

Here, $c_{2}$ propels the particle towards the current best particle within the corresponding sub-swarm of this particle. The most popular local-best swarm topologies include:

1. *Ring topology:* Each particle is affected by its *k* immediate neighbors.
2. *Star/Wheel topology:* Only one particle is in the center of the swarm, and this particle is influenced by all other particles. However, each of the other particles can only access the information of the central particle.
3. *Pyramid topology:* The swarm of particles are divided into several levels, and there are $4^{l}$ particles in level *l* (l≥0), which form a $2^{l}\times 2^{l}$ mesh.
4. *Von Neumann topology:* All particles are connected as a toroidal, and each particle has four neighbors, which are above, below, left, and right particles.
5. *Random topology:* Each particle chooses neighbors randomly at each iteration. We utilize the second algorithm proposed in [24] to generate the random topology.

### 2.3. Evaluation Criteria of PSO-Based Localization Algorithms

1. *Localization error.* The localization error of unknown node $U_{j}$ is defined as $E_{j}=\frac{1}{R}\sqrt{{\left({\hat{x}}_{u_{j}}-x_{u_{j}}\right)}^{2}+{\left({\hat{y}}_{u_{j}}-y_{u_{j}}\right)}^{2}}$. The mean and standard deviation of localization error are denoted by $\bar{E}$ and $\sigma_{E}$, respectively.
2. *Number of iterations.* This is the number of iterations of PSO to achieve the best fitness. The mean and standard deviation of the number of iterations are denoted by $\bar{Itr}$ and $\sigma_{Itr}$, respectively.

In WSN applications, the localization error depends on ranging error, GPS error, localization error accumulation, and the localization algorithm. The ranging error results from the distance measurement technique, and the GPS error determines the errors of anchors’ positions. Both ranging and GPS errors are assumed to obey Gaussian distribution, and they are denoted by *e*. The accumulated localization error comes from the iterative localization procedure: some unknown nodes may utilize localized unknown nodes to localize themselves, while the positions of these localized unknown nodes already have localization error.

## 3. A Survey of PSO-Based Localization Algorithms

### 3.1. Basic Procedure of PSO-Based Localization Algorithms

After measuring the distance between sensor nodes, each unknown node estimates its location by Algorithm 1. In this algorithm, Line 1 determines the particles’ search space, which is the intersection region of the radio range of all neighboring reference nodes of this sensor node. After initialization in Line 2, it uses an iterative process to estimate the position (Lines 3 to 7). Note that the update process of Line 6 is different according to different PSO-based localization algorithms.

**Algorithm 1** PSO-Based Localization Algorithm1:Determine the particles’ search space.2:Initialize a swarm of particles in the search space with random positions and velocities.3:**while** stop criteria are not met **do**4: Compute the fitness values of all particles.5: Compute $Q_{i}$, $Lb_{i}$, and/or Gb.6: Update each particle.7:**end** **while**

### 3.2. PSO-Based Localization Algorithms

The basic PSO algorithm using (8) is the most popular one among all PSO-based localization algorithms, and we use WPSO (weighted-PSO) to represent it for convenience.

The first WPSO-based localization algorithm was proposed in [25], which uses (6) as the objective function. WPSO is also applied to the localization problem of ultra-wide band sensor networks in [26,27]. In a static sensor network, some unknown nodes may not have enough neighbor anchors to localize themselves, so [28] applies DV (Distance Vector)-Distance to make all unknown nodes have distances to at least three anchors, and then it uses WPSO to localize. In order to make WPSO’s convergence rate fast, [29] introduces a threshold to constrain a change of fitness function. Due to the inaccurate distance measurements, flip ambiguity is popular during localization, but this problem is not considered by the aforementioned algorithms. In [30], WPSO in conjunction with two types of constraints is used to cope with this problem. Besides two-dimensional sensor networks, WPSO is also applied to localize three-dimensional WSNs [27,31,32] and underwater WSNs [33]. Different from the above algorithms, a mobile anchor-assisted WPSO-based localization algorithm is proposed in [20,34], which only uses one mobile anchor to provide distance range to all unknown nodes while it traverses the sensor network. Besides localization, [35] also applies WPSO to the real-time autonomous deployment of sensor nodes (including anchors and unknown nodes) from an unmanned aerial vehicle.

Besides WPSO, many variants of PSO algorithms have been proposed to improve the performance. The most representative algorithms are listed below.

1. *Constricted PSO (CPSO)*. WPSO has the disadvantages of early convergence and swarm explosion, so CPSO [36] introduces the constriction coefficient *χ* to conquer these disadvantages:
  (11) $$\begin{array}{cc} \begin{array}{c} V_{ij}\;=\;\chi \left(V_{ij}\;+\;\varphi_{1j}\left(Q_{ij}\;-\;P_{ij}\right)\;+\;\varphi_{2j}\left(Gb_{j}\;-\;P_{ij}\right)\right)\\ P_{ij}=P_{ij}+V_{ij} \end{array} & j=1,2, \end{array}$$
  where $\chi =\frac{2}{\left|2-c-\sqrt{c^{2}-4c}\right|}$, $c=c_{1}+c_{2}>4.0$. CPSO eliminates the $V_{max}$ in WPSO. It performs better than WPSO in many problems [36].
2. *H-Best PSO (HPSO)*. Global- and local-best PSO algorithms have their own advantages and disadvantages. Combining these two algorithms, HPSO [9,14] divides the particles into several groups, and particle *i* is updated based on Gb, $Lb_{i}$, and $Q_{i}$, per the following equation:
  (12) $$\begin{array}{cc} \begin{array}{c} V_{ij}=\omega V_{ij}+c_{1}\varphi_{1j}\left(Q_{ij}-P_{ij}\right)+c_{2}\varphi_{2j}\left(Gb_{j}\;-\;P_{ij}\right)\;+\;c_{3}\varphi_{3j}\left(Lb_{ij}\;-\;P_{ij}\right)\\ P_{ij}=P_{ij}+V_{ij} \end{array} & j=1,2. \end{array}$$
  Here, $c_{3}$ is the same as $c_{2}$ of (10), and $\varphi_{3j}$ is a random number uniformly distributed in (0,1). HPSO provides fast convergence and swarm diversity, but it utilizes more parameters than WPSO.
3. *PSO with particle permutation (PPSO)*. In order to speed up the convergence, PPSO [37] sorts all particles such that $f\left(P_{i}\right)\leq f\left(P_{j}\right)$, if i≤j, and replaces the positions of particles $\lfloor \frac{M}{2}\rfloor +1$ to *M* with positions close to $P_{1}$. The rule of replacement is:
  (13) $$\begin{array}{ccc} P_{\lfloor \frac{M}{2}\rfloor +k,j}=P_{1j}+\rho_{kj} & j=1,2; & k=1,2,\cdots ,\lceil \frac{M}{2}\rceil \;, \end{array}$$
  where $\rho_{kj}$ is a random number uniformly distributed in (−0.5,0.5).
4. *Extremum disturbed and simple PSO (EPSO)*. Sometimes, PSO easily fall into local extrema. EPSO [38] uses two preset thresholds $T_{0}$ and $T_{g}$ to randomly churn $Q_{i}$ and Gb to overcome this shortcoming. The operators of extremal perturbation are:
  (14) $$\begin{array}{ccc} Q_{ij}^{\prime }=\varphi_{4j}Q_{ij}, & Gb_{j}^{\prime }=\varphi_{5j}Gb_{j} & j=1,2. \end{array}$$
  Let $t_{0}$ and $t_{g}$ be evolutionary stagnation iterations of $Q_{i}$ and Gb, respectively. In (14), if $t_{0}\leq T_{0}$ ($t_{g}\leq T_{g}$), $\varphi_{4j}$ ($\varphi_{5j}$) is 1; Otherwise, $\varphi_{4j}$ ($\varphi_{5j}$) is a random number uniformly distributed in [0,1]. For particle *i*, the update function of EPSO is
  (15) $$\begin{array}{cc} P_{ij}=\eta_{ij}\omega P_{ij}\;+\;c_{1}\varphi_{1j}\left(Q_{ij}^{\prime }\;-\;P_{ij}\right)\;+\;c_{2}\varphi_{2j}\left(Gb_{j}^{\prime }-P_{ij}\right) & j=1,2, \end{array}$$
  where $\eta_{ij}$ is used to control the movement direction of particle *i* to make the algorithm convergence fast, which is defined as
  (16) $$\begin{array}{cc} \eta_{ij}=\frac{P_{ij}-Gb_{j}}{\sqrt{{\left(P_{i1}-Gb_{1}\right)}^{2}+{\left(P_{i2}-Gb_{2}\right)}^{2}}} & j=1,2. \end{array}$$
5. *Dynamic PSO (DPSO)*. Each particle in DPSO [19] pays full attention to the historical information of all neighboring particles, instead of only focusing on the particle which gets the optimum position in the neighborhood. For particle *i*, the update function of DPSO is
  (17) $$\begin{array}{cc} P_{ij}=P_{ij}+c_{1}\left(P_{ij}-P_{ij}^{\prime }\right)+\frac{c_{2}}{K}\sum_{k=1}^{K}\left(Q_{kj}-P_{ij}\right)+\frac{c_{3}\varphi_{3j}}{K}\sum_{k=1}^{K}|Q_{kj}-Q_{ij}| & j=1,2\;, \end{array}$$
  where *K* is the number of neighboring particles of the *i*th particle. $P_{i}^{\prime }$ is the previous position of the *i*th particle. Note that $c_{1}$, $c_{2}$, and $c_{3}$ are just weights without physical meaning.
6. *Binary PSO (BPSO)*. BPSO [39] is used in binary discrete search space, which applies a sigmoid transformation to the speed attribute in update function, so its update function of particle *i* is
  (18) $$\begin{array}{cc} \begin{array}{c} V_{ij}=\omega V_{ij}\;+\;c_{1}\varphi_{1j}\left(Q_{ij}\;-\;sigmoid\left(P_{ij}\right)\right)+\;c_{2}\varphi_{2j}\left(Gb_{j}-sigmoid\left(P_{ij}\right)\right)\\ P_{ij}=P_{ij}+V_{ij} \end{array} & j=1,2, \end{array}$$
  where $sigmoid(P_{ij})$ is defined as
  (19) $$sigmoid\left(P_{ij}\right)\;=\;\left\{\begin{array}{cc} 1 & if\;\varphi_{6}\;<\;2\left|\frac{1}{1\;+\;e^{-V_{ij}}}\;-\;0.5\right|\\ 0 & otherwise \end{array}\right.$$
  $\varphi_{6}$ is a random number.
7. *PSO with time variant ω, $c_{1}$, and $c_{2}$ (TPSO)*. TPSO [19] employs time-varying $c_{1}$, $c_{2}$, and *ω* (see Equation (9)) to achieve proper balance between global and local exploitation, where
  (20) $$c_{1}=\left(c_{1f}-c_{1s}\right)\frac{t}{t_{max}}+c_{1s},\;c_{2}=\left(c_{2f}-c_{2s}\right)\frac{t}{t_{max}}+c_{2s}\;,$$
  where $c_{1s}$, $c_{1f}$, $c_{2s}$, and $c_{2f}$ are *initial* and *final* values of $c_{1}$ and $c_{2}$, respectively.
8. *PSO with particle migration (MPSO)* [40]. MPSO enhances the diversity of particles and avoids premature convergence. MPSO randomly partitions particles into several sub-swarms, each of which evolves based on TPSO, and some particles migrate from one sub-swarm to another during evolution.

In one word, the above-mentioned PSO variants aim to overcome one or more drawbacks of WPSO, but they also introduce additional operations.

### 3.3. Comparison between PSO and Other Optimization Algorithms

The above-mentioned algorithms are also compared with the other algorithms in corresponding references, and Table 1 summarizes the comparison results.

The bacterial foraging algorithm models the foraging behavior of bacteria that thrive to find nutrient-rich locations. Each bacterium moves using a combination of tumbling and swimming. Tumbling refers to a random change in the direction of movement, and swimming refers to moving in a straight line in a given direction.

The simulated annealing algorithm originated from the formation of crystals from liquids. Initially, the simulated annealing algorithm is in a high energy state. At each step, it considers some neighbouring state of the current state, and probabilistically decides between moving the system to one neighbouring state or staying in the current state. These probabilities ultimately lead the system to move to states of lower energy.

Artificial neural networks model the human brain in performing an intelligent task. It integrates computational units (neurons) in multi-layers, and these layers are connected by adjustable weights. Three traditional layers are input, hidden, and output.

The biogeography-based optimization algorithm is motivated by the science of biogeography, which investigates the species distribution and its dynamic properties from past to present spatially and temporally. In this algorithm, the candidate solutions and their features are considered as islands and species, respectively. Species migrate among islands, which is analogous to candidate solutions’ interaction.

## 4. Parameter Selections of PSO-Based Localization Algorithms

Given a problem to be solved, the performance of a PSO depends on its parameters. Although theoretical analysis can guide the parameter selection, this analysis can occupy large space, such as [43], which exceeds the limit of this paper. On the other hand, extensive experimentation has been used widely in the parameter selection or performance analysis of PSO [15,21,23]. Therefore, we try to choose the best parameter by experiments instead of theoretical analysis. Because some parameters of HPSO and PPSO have been calibrated in corresponding references, we only calibrate parameters of WPSO, CPSO, MPSO, TPSO, BPSO, EPSO, and DPSO, and *M* and $V_{max}$ of PPSO and HPSO.

### 4.1. Simulation Setup

The PSO-based localization algorithms are implemented in C language, and the results are analyzed by Matlab. Because our aim is to decide the best parameters, we only perform the localization procedure on one unknown node, denoted by $U_{1}$. Suppose the actual position of $U_{1}$ is $\left(x_{u_{1}},y_{u_{1}}\right)=\left(0,0\right)$, and $N_{B}$ reference nodes are deployed within the communication range of $U_{1}$. Considering the error *e* during distance measurements, ${\hat{d}}_{k1}$ ($k=1,2,\cdots ,N_{B}$) is defined as

(21) $${\hat{d}}_{k1}=d_{k1}+\alpha \;,$$

where $d_{k1}$ is the real distance between $U_{1}$ and $B_{k}$, *α* is a random number that follows a normal distribution with mean 0 and variance $d_{k1}e$.

The simulation setup is shown in Table 2. For convenience, we use *a*:*b*:*c* to represent the set of [a,a+b,⋯,c] in this paper. The parameter selection procedure is:

1. Using $V_{max}=0.5R$ and M=20,30,40,50 to find out the best $c_{1}$, $c_{2}$, $c_{3}$, and *ω*.
2. Using the best $c_{1}$, $c_{2}$, $c_{3}$ and *ω* to choose *M*.
3. Using the best $c_{1}$, $c_{2}$, $c_{3}$, *ω* and *M* to choose $V_{max}$.
4. Using the best $c_{1}$, $c_{2}$, $c_{3}$, *ω*, *M* and $V_{max}$ to compare fitness functions.

We use $f_{3}$ as the objective function of PSO to choose the best parameters, because it is the most popular fitness used in PSO-based localization algorithms. The stopping criteria is

(22) $$f_{3}\left(X\right)<=10^{-4}\vee t>t_{max}\;,$$

which means the fitness value $f_{3}\left(X\right)$ achieves an allowable precision $10^{-4}$ or the number of iteration *t* exceeds pre-defined threshold $t_{max}$.

We generate 100 tests with each combination of different *e*, *R*, and $N_{B}$, and we utilize each algorithm to estimate the position of $U_{1}$ by 100 runs for each test case, under each group of PSO’s parameters. The results are the average of these runs. A localization algorithm should have the best performance regardless of $N_{B}$, because $N_{B}$ is different to each unknown node in real applications of WSN. Hence, the impacts of $N_{B}$ on parameter selection are not analyzed.

### 4.2. Best Parameters of PSO-Based Localization Algorithms

**Figure 1:**
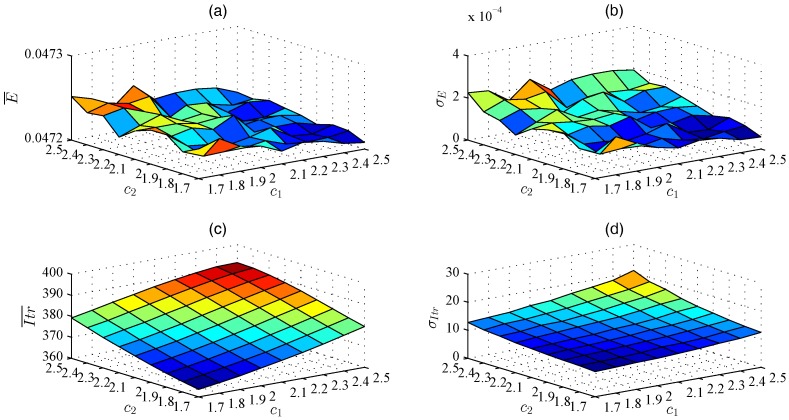
Localization performance of different $c_{1}$ and $c_{2}$ using WPSO with global-best model. (**a**) $\bar{E}$; (**b**) $\sigma_{E}$; (**c**) $\bar{Itr}$; (**d**) $\sigma_{Itr}$.

Taking the selection of $c_{1}$ and $c_{2}$ of WPSO with global-best model as an example, the selection approach is introduced. We first calibrate $c_{1}$ and $c_{2}$ without considering the specific value of *e*, because we may not know *e* during localization. The results are the averages of all *e*. Figure 1a,b show that the impacts of $c_{1}$ and $c_{2}$ on localization errors are very little: the gap between the minimum and maximum $\bar{E}$ ($\sigma_{E}$) is about $4.42\times 10^{-5}$ ($1.79\times 10^{-4}$). On the contrary, Figure 1c,d show that $\bar{Itr}$ and $\sigma_{Itr}$ reduce with the decrease of $c_{1}$ and $c_{2}$. With different $c_{1}$ and $c_{2}$, the difference between the maximum and minimum $\bar{Itr}$ ($\sigma_{Itr}$) is about 31.03 (11.70). The best choices are $c_{1}=$ 1.7–1.8 and $c_{2}=$ 1.7–1.8, which occupy 96.2% optimal number of iterations for all cases. Further, we investigate the impacts of $c_{1}$ and $c_{2}$ on localization performance under each *e*, and we find that $c_{1}=$ 1.7–1.8 and $c_{2}=$ 1.7–1.8 are still the best choice for each *e*.

Similar approaches have been applied to other parameters and algorithms, and the resulting best parameters are shown in Table 3. Because MPSO and HPSO already divide the swarm of particles into several sub-swarms which are similar with local-best models, we only analyze their global-best models.

### 4.3. Performance Comparison of Fitness Function

**Figure 2:**
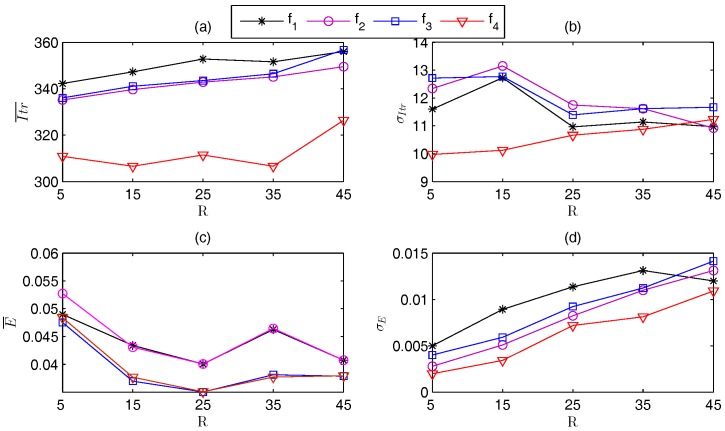
Localization performance of different *f* using WPSO with global-best model.(**a**) $\bar{Itr}$; (**b**) $\sigma_{Itr}$; (**c**) $\bar{E}$; (**d**) $\sigma_{E}$.

Figure 2a,b shows that $\bar{Itr}$ of $f_{4}$ is [306.86, 326.41], which is about 89.65%–91.57% of those of $f_{1}$, $f_{2}$, and $f_{3}$. Figure 2c,d shows that $f_{4}$ is still the best. In detail, $f_{3}$ and $f_{4}$ has almost the same $\bar{E}$, which is [0.035, 0.048]. However, $f_{4}$ has the minimal $\sigma_{E}$.

The performance of each fitness function using the other PSO algorithms with different swarm topologies also shows that $f_{4}$ outperforms the other functions in all cases.

## 5. Performance Comparisons of PSO-Based Localization Algorithms

### 5.1. Simulations Setup

The performance of all PSO algorithms is compared by simulations of a whole WSN. The parameters of each PSO variant are set based on Section 4. During localization, we utilize iterative localization to localize as many unknown nodes as possible. We also demonstrate the impact of radio irregularity on the localization performance of different algorithms, because the actual transmission range of sensor nodes is not a perfect circle due to multi-path fading, shadowing, and noise. The radio model in [44] is used to represent the degree of irregularity *D*. Based on this model, there is an upper bound *R* and a lower bound (1−D)R of the communication range. The simulation setups are: L= 100 units, $N_{U}=$ 100:100:500, $N_{A}=$ 10:10:50, R= 25:10:45 units, e= 0:0.05:0.2, D= 0:0.1:0.5.

### 5.2. Comparisons of Different Swarm Topologies in Same PSO

**Figure 3:**
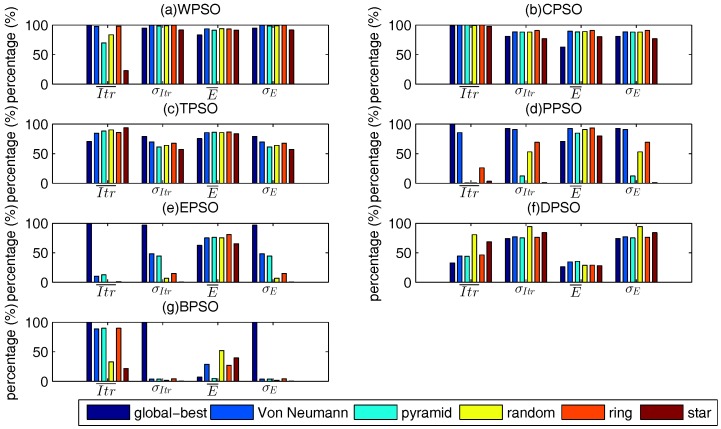
Percentage of each swarm topology achieving the optimal values. (**a**) WPSO; (**b**) CPSO; (**c**) TPSO; (**d**) PPSO; (**e**) EPSO; (**f**) DPSO; (**g**) BPSO.

There are 2250 test cases to simulate, and the best swarm topology should outperform the other ones in most test cases, instead of in several test cases. Therefore, we count the number of optimal values of each swarm topology of all test cases, and try to find the topology which achieves the optimal values in most test cases. The “optimal values” are the evaluation criteria mentioned in Section 2.3. The results are shown in Figure 3.

For WPSO, Von Neumann and ring topologies perform almost the same, and they outperform the other topologies. Ring topology can gain the optimal $\bar{E}$, $\sigma_{E}$, $\bar{Itr}$, and $\sigma_{Itr}$ in more than 93.2%, 99.5%, 98.1%, and 99.5% of test cases, respectively, and Von Neumann topology can gain the optimal $\bar{E}$, $\sigma_{E}$, $\bar{Itr}$, and $\sigma_{Itr}$ in more than 93.4%, 99.5%, 97.5%, and 99.5% of test cases, respectively.

It is hard to say which topology is the best for some algorithms, because none of these topologies performs the best in all four percentages. In this case, we give more importance to $\bar{E}$ than the other criteria. Taking PPSO as an example (Figure 3d), its global-best and Von Neumann topologies are obviously better than the others, but Von Neumann outperforms global-best model in $\bar{E}$, while global-best model is better than Von Neumann in the other three criteria. Since we put $\bar{E}$ as the first criterion to choose the best swarm topology, Von Neumann is the best one which achieves optimal $\bar{E}$ in more than 92.3% cases, while the percentage of global best model is 70.7%.

Using a similar idea, the best swarm topologies of all algorithms are: ring topology for WPSO, CPSO, and TPSO; Von Neumann topology for PPSO and DPSO; random topology for EPSO and BPSO.

Furthermore, we compare the performance of different swarm topologies used in the same PSO algorithm, with different $N_{U}$, $N_{A}$, *R*, *D*, *e* one by one, and we find these results are consistent with the aforementioned results.

### 5.3. Comparison of PSO-Based Localization Algorithms

Using the results introduced in Section 4, the PSO-based localization algorithms are compared.

#### 5.3.1. General Analysis

**Figure 4:**
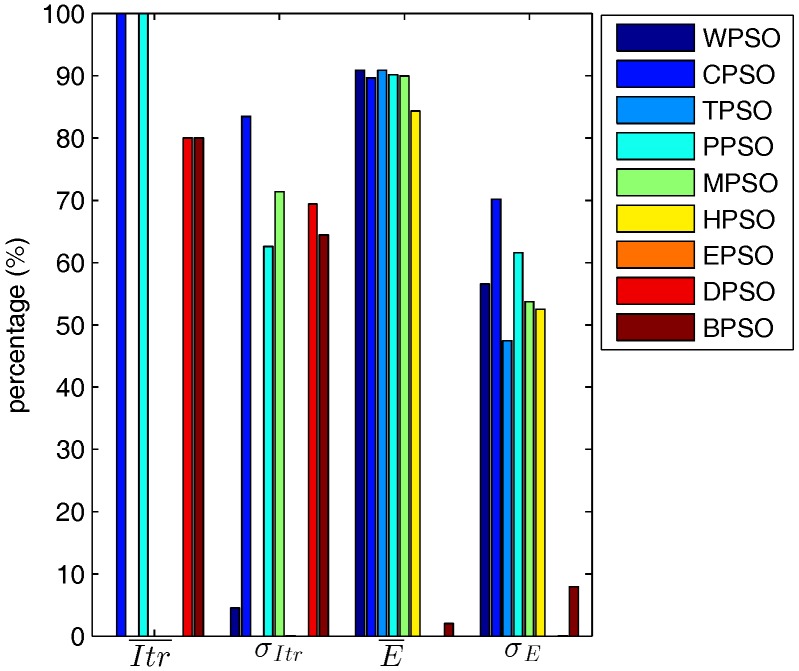
Percentage of PSO-based localization achieving the optimal values.

In general, as shown in Figure 4, CPSO and PPSO needs fewer iterations than the other PSO-based localization algorithms. In fact, the average $\bar{Itr}$ of all test cases required by CPSO and PPSO are 220.16 and 222.29, respectively, while those of WPSO, TPSO, MPSO, HPSO, EPSO, DPSO, and BPSO are 312.88, 484.99, 268.84, 281.68, 310.26, 253.52, and 252.47, respectively. $\sigma_{Itr}$ of CPSO is also the smallest (6.67), while $\sigma_{Itr}$ of the other algorithms are larger than 10. On the other hand, CPSO and PPSO achieve the optimal $\bar{E}$ for more than 89.69% of test cases, and the optimal $\sigma_{E}$ for more than 60.84% of test cases. Furthermore, the average $\bar{E}$ and $\sigma_{E}$ of all test cases of CPSO and PPSO are the first two best ones among all algorithms. CPSO has the least operations during one iteration among all algorithms. Compared with CPSO, the other algorithms need more parameters and operations such, as $V_{max}$, particle sort, or migration.

In one word, CPSO is the best PSO-based localization algorithm.

#### 5.3.2. Impacts of Network Parameters on Performance

**Figure 5:**
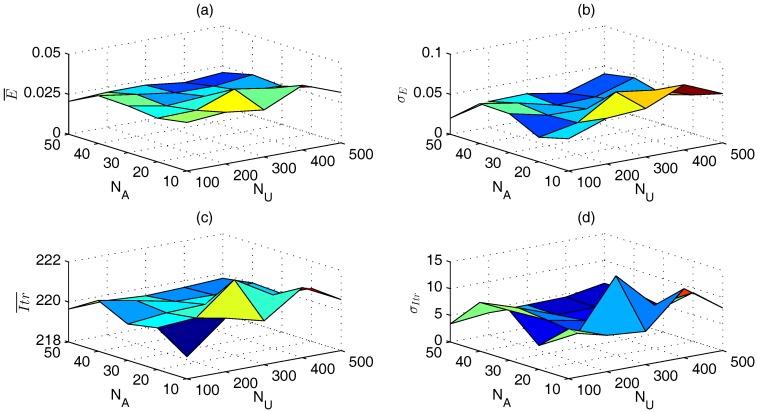
Impacts of $N_{U}$ and $N_{A}$ on the localization performance of CPSO. (**a**) $\bar{E}$; (**b**) $\sigma_{E}$; (**c**) $\bar{Itr}$; (**d**) $\sigma_{Itr}$.

**Figure 6:**
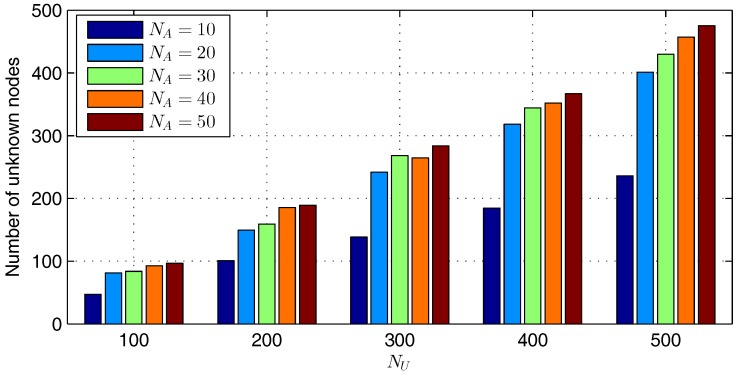
Number of unknown nodes localized by neighboring anchors under different $N_{U}$ and $N_{A}$ with CPSO.

In order to analyze the impacts of network setups on localization performance, CPSO is taken as an example, because we find that the other algorithms have the same trends as CPSO, except the variation range. As illustrated in Figure 5, we cannot draw any rule of the impacts of $N_{U}$ and $N_{A}$ on $\bar{Itr}$, $\sigma_{Itr}$. However, $\bar{E}$ and $\sigma_{E}$ decrease as $N_{A}$ increases, because the more anchors exist in a WSN, the less unknown nodes need iterative localization, as shown in Figure 6. Figure 6 shows that the number of unknown nodes localized by neighboring anchors instead of localized unknown nodes under $N_{A}=500$ is about two times the number under $N_{A}=100$.

Table 4 denotes that the variations are very small except $\sigma_{Itr}$. $\bar{E}$ and $\sigma_{E}$ are almost the same, but CPSO and PPSO have the smallest $\bar{Itr}$.

**Figure 7:**
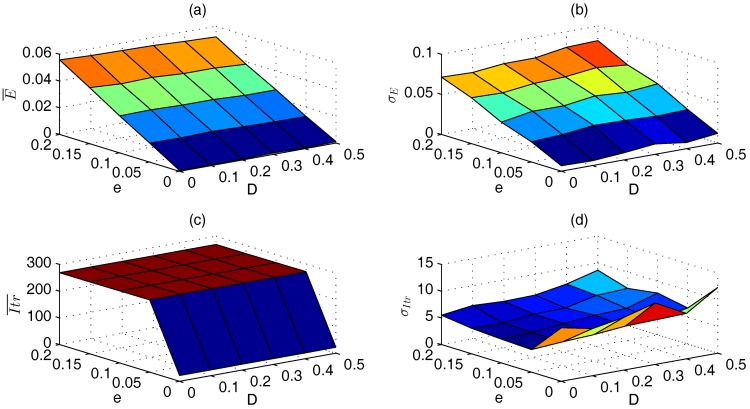
Impacts of *D* and *e* on localization performance of CPSO. (**a**) $\bar{E}$; (**b**) $\sigma_{E}$; (**c**) $\bar{Itr}$; (**d**) $\sigma_{Itr}$.

From Figure 7a,b, we can see that *D* impacts the localization error very little, where $\bar{E}$ ($\sigma_{E}$) only differs less than 0.003 (0.006) with the same *e* and different *D*. However, *e* affects the localization error greatly: a larger *e* leads to a larger localization error. As shown in Figure 7c, $\bar{Itr}$ is minimal when D=e=0, and it increases significantly when both *D* and *e* are greater than 0. However, it keeps almost the same when D>0 and e>0, which can also be proved from Table 5. On the contrary, $\sigma_{Itr}$ reaches maximum when D=e=0, and it decreases when both *D* and *e* are greater than 0, as shown in Figure 7d.

Table 5 shows that $\bar{E}$ and $\sigma_{E}$ of EPSO, DPSO, and BPSO are larger than the other algorithms, and *D* and *e* have little impacts on $\bar{Itr}$, but $\sigma_{Itr}$ varies very much under different *D* and *e*.

**Figure 8:**
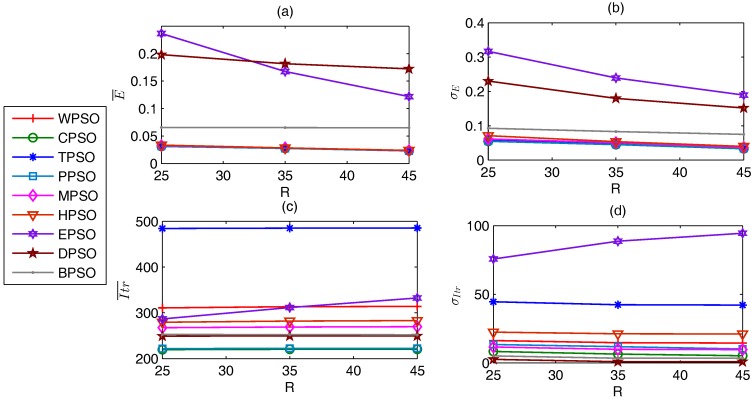
Impacts of *R* on localization performance. (**a**) $\bar{E}$; (**b**) $\sigma_{E}$; (**c**) $\bar{Itr}$; (**d**) $\sigma_{Itr}$.

Figure 8a,b denote that the larger *R* has higher localization precision. $\bar{E}$ of EPSO, DPSO, and BPSO are larger than the other algorithms, while the other algorithms have almost the same $\bar{E}$. Moreover, $\bar{E}$ of EPSO and DPSO decrease with increasing *R*, while those of the other algorithms show little change. $\sigma_{E}$ has the same rule as $\bar{E}$, as shown in Figure 8b. Figure 8c,d show that the impacts of *R* on the number of iterations are very little, and CPSO and PPSO requires the fewest iterations, while TPSO needs the most iterations.

### 5.4. Comparison between CPSO and SOCP

Table 1 shows the comparison between PSO and the other optimization algorithms. We compare CPSO and second-order cone programming (SOCP) in this section, because SOCP is also a popular optimization algorithm used in localization problems [4,5], and CPSO has not been compared with SOCP. The SOCP algorithm is implemented in Matlab by CVX [45].

**Figure 9:**
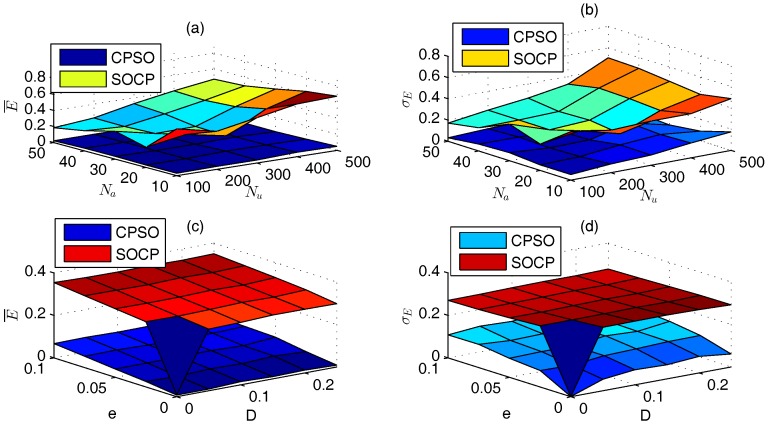
Comparison of CPSO and SOCP. (**a**) $\bar{E}$ of different $N_{U}$ and $N_{A}$; (**b**) $\sigma_{E}$ of different $N_{U}$ and $N_{A}$; (**c**) $\bar{E}$ of different *D* and *e*; (**d**) $\sigma_{E}$ of different *D* and *e*.

As shown in Figure 9, CPSO with global-best model outperforms SOCP under different $N_{U}$, $N_{A}$, *D*, and *e*. $\bar{E}$ and $\sigma_{E}$ of CPSO are 0.0085–0.0684 and 0.0526–0.1311, respectively, and those of SOCP are 0.2909–0.3549 and 0.2707–0.3, respectively. Further, SOCP takes much longer than CPSO. For example, there are 450 test cases when $N_{U}=100$, and SOCP uses 2 hours and 58 minutes to obtain the results, while CPSO only takes 12 minutes.

## 6. Conclusions

As a classical swarm intelligence algorithm, particle swarm optimization has many advantages over other optimization algorithms to solve the localization problem of wireless sensor networks, and many particle swarm optimization-based localization algorithms have been proposed in recent years, but it lacks of parameter selection and comprehensive comparison of these algorithms. This paper surveys the existing particle swarm optimization-based localization algorithms, and chooses the best parameters based on simulations. Further, we compare currently widely-used particle swarm optimization-based localization algorithms with six types of swarm topologies, and the results show that particle swarm optimization with constriction coefficient and ring topology is the best choice to solve the localization algorithm of wireless sensor networks.

## Figures and Tables

**Table 1:** Advantages of particle swarm optimization (PSO)-based localization algorithms. HPSO: H-Best PSO; PPSO: PSO with particle permutation; WPSO: weighted-PSO.

Algorithm	References	Comparative	Advantages of PSO
WPSO	[12,35]	bacterial foraging algorithm	faster
WPSO	[25]	simulated annealing	more accurate
WPSO	[41]	Gauss–Newton algorithm	more accurate
WPSO	[26,31]	least square	more accurate
WPSO	[42]	simulated annealing, semi-definite programming	faster, more accurate
WPSO	[28]	artificial neural network	more accurate
WPSO	[32]	least square	faster, more accurate
HPSO	[9,14]	biogeography-based optimization	faster
PPSO	[37]	two-stage maximum-likelihood, plane intersection	faster, more accurate

**Table 2:** Simulations setup. CPSO: Constricted PSO; DPSO: Dynamic PSO; EPSO: Extremum disturbed and simple PSO; MPSO: PSO with particle migration; TPSO: PSO with time variant *ω*, $c_{1}$, and $c_{2}$.

Type	Values
network	*R* = 5:10:45, *e* = 0.05:0.05:0.2, $N_{B}$ = 3:20
General ${}^{a}$	$V_{max}$ = 0.1*R*:0.1*R*:2*R*, *M* = 5:5:100, $t_{max}$ = 500
WPSO, EPSO	$\omega_{max}$ = 1.4:−0.1:0.9, $\omega_{min}$ = 0.4:−0.1:0, $c_{1}$ = 1.7:0.1:2.5, $c_{2}$ = 1.7:0.1:2.5
CPSO	$c_{1}$ = 2.05:0.05:2.5, $c_{2}$ = 2.05:0.05:2.5
MPSO, TPSO	$\omega_{max}$ = 1.4:−0.1:0.9, $\omega_{min}$ = 0.4:−0.1:0, $c_{1i}$ = $c_{2f}$ = 3:−0.25:0.25, $c_{2i}$ = $c_{1f}$ = $c_{1}$−0.25:−0.25:0.25
HPSO	*ω* = 0.7, $c_{1}$ = $c_{2}$ = $c_{3}$ = 1.494
PPSO	*ω* = 1, $c_{1}$ = $c_{2}$ = 2.0
BPSO	*ω* = 1.0:−0.1:0, $c_{1}$ = 1.7:0.1:2.5, $c_{2}$ = 1.7:0.1:2.5
DPSO	$c_{1}$ = 1.0:0.1:0.1, $c_{2}$ = 2.5:−0.1:1.5, $c_{3}$ = 0:0.1:1.0

${}^{a}$ The parameters used by all PSO variants.

**Table 3:** Parameter selections of PSO-based localization algorithms. BPSO: Binary PSO.

Variant	Topology	$\left[c_{1},c_{2},c_{3}\right]$	$\left[\omega_{\max},\omega_{\min}\right]$	*M*	$V_{\max}$
WPSO	global-best	[1.7–1.8,1.7–1.8,—]	[0.9,0]	30	0.1R
	pyramid	[1.7,1.7–1.8,—],[1.8,1.7,—]	[0.9,0]	21	0.1R
	random	[1.7,1.7–1.8,—]	[0.9,0]	30	0.1R
	Von Neumann	[1.7,1.7–1.8,—],[1.8,1.7]	[0.9,0]	25	0.1R
	ring	[1.7,1.7–1.8,—],[1.8,1.7,—]	[0.9,0]	25	0.1R
	star	[1.7,1.7–1.8,—],[1.8,1.7,—]	[0.9,0]	25	0.1R
CPSO	global-best	[2.4,2.5,—],[2.45,2.45–2.5,—]	—	10	—
	pyramid	[2.45,2.5,—],[2.5,2.45–2.5,—]	—	21	—
	random	[2.5,2.5,—]	—	10	—
	Von Neumann	[2.45,2.45–2.5,—],[2.5,2.4–2.5,—]	—	10	—
	ring	[2.45,2.5,—],[2.5,2.4–2.5,—]	—	10	—
	star	[2.4,2.5,—],[2.45,2.45–2.5,—]	—	10	—
TPSO	global-best	[0.5,0.25,—],[0.75–1,0.25–0.5,—]	[0.9,0]	25	0.1R
	pyramid	[0.5–1,0.25,—],[0.75,0.25–0.5,—]	[0.9,0]	21	0.1R
	random	[0.5,0.25,—],[0.75–1,0.25–0.5,—]	[0.9,0]	30	0.1R
	Von Neumann	[0.5–1.25,0.25,—]	[0.9,0]	25	0.1R
	ring	[0.75–1.25,0.25–0.5,—]	[0.9,0]	25	0.1R
	star	[0.5–1,0.25–0.25,—]	[0.9,0]	15	0.1R
PPSO	all	[2.0,2.0,—]	*ω* = 1.0	20-40	0.1R
EPSO	global-best	[2.5,1.7,—]	[1.4,0.4]	45	0.1R
	pyramid	[2.5,1.8,—]	[1.4,0.4]	21	0.1R
	random	[2.4,2.5,—]	[0.9,0]	20	0.1R
	Von Neumann	[2.4,2.5,—]	[0.9,0]	30	0.1R
	ring	[2.4,2.5,—]	[0.9,0]	25	0.1R
	star	[2.4,2.5,—]	[0.9,0]	20	0.1R
DPSO	global-best	[0.7/0.8,2.1,0.4/0.5]	—	75	0.1R
	pyramid	[0.7/0.8/0.9,2.3,0.4]	—	21	0.1R
	random	[0.9,2.3,0.4]	—	30	0.1R
	Von Neumann	[0.7/0.8/0.9,2.3,0.4]	—	35	0.1R
	ring	[0.7/0.8/0.9,2.3,0.4]	—	35	0.1R
	star	[0.7/0.8,2.1,0.4/0.5]	—	45	0.1R
BPSO	global-best	[2.1/2.2,1.7,—]	*ω* = 1.4/1.5	75	0.1R
	pyramid	[2.3/2.4,1.7,—]	*ω* = 1.4	21	0.1R
	random	[1.9,2.0,—]	*ω* = 1.3/1.4/1.5	75	0.1R
	Von Neumann	[2.0–2.3,1.7,—]	*ω* = 1.5	80	0.1R
	ring	[2.1/2.2,1.7,—]	*ω* = 1.4/1.5	35	0.1R
	star	[2.5,1.9,—]	*ω* = 1.3	80	0.1R
MPSO	global-best	[1/1.25,0.25/0.5,—],[1.5,0.25,—]	[0.9,0]	45	0.1R
HPSO	global-best	[1.494,1.494,1.494]	*ω* = 0.7	45	0.1R

**Table 4:** Variation range of different $N_{U}$ and $N_{A}$.

Criteria	WPSO	CPSO	TPSO	PPSO	MPSO	HPSO	EPSO	DPSO	BPSO
$\bar{Itr}$	311.6–314.9	219.1–221.8	482.4–485.8	220.8–225.6	267.8–270.6	279.6–285.5	304.6–316.6	252.2–254.7	251.1–252.7
$\sigma_{Itr}$	13.91–19.96	3.39–14.92	40.64–50.77	7.86–20.34	9.06–14.57	17.94–27.31	83.20–90.10	0.78–16.83	3.38–19.27
$\bar{E}$	0.02–0.04	0.02–0.04	0.02–0.044	0.03–0.05	0.02–0.05	0.02–0.04	0.14–0.27	0.17–0.23	0.06–0.08
$\sigma_{E}$	0.02–0.09	0.02–0.08	0.02–0.09	0.02–0.08	0.03–0.10	0.02–0.10	0.14–0.41	0.10–0.29	0.06–0.13

**Table 5:** Variation range of different D>0 and e>0.

Criteria	WPSO	CPSO	TPSO	PPSO	MPSO	HPSO	EPSO	DPSO	BPSO
$\bar{Itr}$	359.7–366.1	269.2–270.6	485.0–486.7	271.0–273.2	316.6–320.9	327.3–335.5	308.6–311.4	269.3–269.7	262.2–262.6
$\sigma_{Itr}$	13.3–17.65	4.62–7.82	39.99–43.6	9.36–13.62	8.68–12.79	18.65–22.84	85.49–86.80	0.89–2.79	3.47–5.34
$\bar{E}$	0.01–0.04	0.01–0.04	0.01–0.04	0.01–0.04	0.01–0.04	0.01–0.04	0.16–0.19	0.17–0.19	0.16–0.17
$\sigma_{E}$	0.03–0.07	0.03–0.07	0.03–0.07	0.03–0.07	0.03–0.08	0.04–0.08	0.22–0.26	0.17–0.21	0.17–0.19
